# Rationalizing sensorimotor deficits in autism spectrum disorder

**DOI:** 10.64898/2026.10.12.758517

**Published:** 2026-10-19

**Authors:** Yizhou Chen, Jean-Paul Noel, Paul Schrater, Dora Angelaki, Xaq Pitkow

## Abstract

Autism spectrum disorder (ASD) is a neurodevelopmental condition characterized by socio-communicative, sensory, and motor anomalies. Recently, a host of probabilistic models have been proposed to account for the varied phenotypes observed in ASD. These computational psychiatry efforts, however, have (1) largely focused on simple tasks that artificially segregate action from perception, and (2) unequivocally assumed that both neurotypical and divergent populations attempt to optimize the same cost function. Here, we ask individuals with ASD and neurotypical (NT) controls to perform a task in virtual reality (VR): they should navigate to, and stop at, the position of briefly presented targets in a closed action-perception loop. First, we demonstrate that while summary task performance is insufficient to distinguish between ASD and NT individuals, data-rich virtual trajectories do differentiate these groups. This suggests that ASD and control participants use distinct navigational strategies and/or cost functions. Next, we leverage the theoretical framework of Inverse Rational Control (IRC) to interpret the sensorimotor anomalies present in ASD. IRC does not assume that agents are optimal, but rational: that is, agents attempt to maximize reward according to an internal model of the task, which may not match the true task and environment. Applying IRC to this VR navigation task revealed both sensory and motor differences between ASD and neurotypical participants. The sensory differences are consistent with Bayesian inference under altered weighting of sensory evidence. The motor differences manifest as more frequent and abrupt actions in ASD, which we explain by a reduced subjective action cost in ASD. Together, these results demonstrate the utility of model-based characterization of psychopathology and suggest that the computational study of ASD should consider not only perceptual anomalies, but also subjective costs.

